# Very High-Power Short-Duration (HPSD) Ablation for Pulmonary Vein Isolation: Short and Long-Term Outcome Data

**DOI:** 10.3390/jcdd9080278

**Published:** 2022-08-18

**Authors:** Sebastian Seidl, Tanja Mülleder, Josef Kaiblinger, Stefan Sieghartsleitner, Jasmina Alibegovic-Zaborsky, Elisabeth Sigmund, Michael Derndorfer, Georg Kollias, Helmut Pürerfellner, Martin Martinek

**Affiliations:** 1 Departement of Cardiology, Ordensklinikum Linz GmbH Elisabethinen, 4020 Linz, Austria; 2 Departement of Cardiology, Göttlicher Heiland Wien, 1170 Vienna, Austria

**Keywords:** atrial fibrillation, pulmonary vein isolation, high power short, duration ablation, prognosis

## Abstract

Background: Circumferential pulmonary vein isolation (PVI) using radiofrequency ablation (RFA) is a standard of care intervention for patients with symptomatic atrial fibrillation (AF). During follow-up, a substantial number of patients need a redo procedure due to reconnections on the basis of insufficient non-transmural ablation lesions. High-power short-duration ablation (HPSD) is expected to create efficient lesions while causing fewer complications than in conventional RFA settings. The aim of this study was to compare one-year outcome data of very HPSD (90 Watt, 4 s) to a strategy using 50 Watt HPSD ablation guided by the CLOSE protocol using the Ablation Index (AI), an arbitrary unit composed of power, contact force and ablation time. Methods: We retrospectively analyzed short and long-term (median follow-up 23.2 ± 9.9 months) outcome data from 52 patients that were scheduled for first-do-symptomatic PVI. A very HPSD ablation protocol with 90 Watt and a 4 s duration cut-off was compared to an HPSD CLOSE approach (50 Watts; AI 550 at the anterior LA wall; AI 400 at the posterior LA wall, the roof and the floor) in terms of freedom from AF recurrence in a long-term electrocardiogram (ECG) over a five days surveillance period. To gain an impression of the subjective sense of wellbeing, the Atrial Fibrillation Effects on QualiTy-of-Life (AFEQT) score was recorded. Results: Overall freedom from AF was found in 81% (90 W 4 s) vs. 87.5% (50 W), (*p* = 0.52). There were 3 AF recurrences during the blanking period (90 W 4 s) vs. 1 (50 W). Within each population, one patient was scheduled for a redo-PVI-procedure. The AFEQT score was in favor of the 90 Watt 4 s approach (86.1 vs. 77.5; *p* = 0.37). Conclusion: Within our relatively small studied population, we found hints that in addition to shortening ablation times and radiation exposure without significantly increasing the rate of relevant intraprocedural complications, very high power short-duration ablation (90 W 4 s) provides comparable efficacy rates after one year.

## 1. Introduction

Atrial fibrillation (AF) is a clinical arrhythmia with a potentially malignant course. In addition to a 1.5–3.5-fold increase in mortality, AF is strongly associated with a significant increase in stroke, congestive heart failure as well as a decrease in quality of life. Furthermore, 10–40% of all AF patients are hospitalized due to their disease at least once per year. Taking that into account, the currently high, and in the future still increasing, AF-prevalence and the resulting individual, health–social and health–economic consequences outline the importance of an optimal therapy strategy [1,2]. Interventional AF ablation is an effective treatment option, and electrical isolation of the pulmonary veins (PVI) has remained at the cornerstone of every ablation procedure up until today [3]. However, pulmonary vein reconnection is frequent and is often the result of catheter instability, tissue edema and a reversible non-transmural injury. High-power short duration (HPSD) RF ablation is a novel technology which is considered to minimize conductive heat transfer and increase resistive heating in order to achieve atrial transmural lesion formation with less edema and more irreversible myocardial tissue injury yet with a reduced risk of collateral tissue damage such as esophageal thermal injury (EDEL) [4,5]. However, little is known about the safety and long-term results of very HPSD (90 Watt 4 s duration protocol) compared to the strategy using 50 Watts guided by the CLOSE protocol using the Ablation Index (AI). Furthermore, the recent clinical practice guidelines for AF recommend an appropriate assessment of the individual’s health-related quality of life (HRQoL) and the requirement to personalize the subsequent management strategy accordingly. The importance of this approach has significantly increased over the years.

Therefore, our study aims to investigate the efficacy and clinical outcome after one year of very HPSD 90 W 4 s ablation in a high-volume single-center experience.

## 2. Methods

This study is a retrospective two-arm non-randomized observational trial comparing the one-year outcome of patients undergoing PVI using an HPSD RF ablation setting. The study has been performed in accordance with the ethical standards as laid down in the Declaration of Helsinki and its later amendments. All patients provided written informed consent for the procedures.

### 2.1. Study Population

We included consecutive patients with symptomatic drug-refractory paroxysmal or persistent AF scheduled for RF-based HPSD AF ablation from July 2020 until December 2020. In addition, the 9 patients from the QDOT-FAST trial who were treated at our institution in May 2018 were included in the analysis [6]. AF was categorized as paroxysmal (lasting < 1 week) or persistent (lasting > 1 week and <1 year or requiring pharmacological or electrical cardioversion in <1 week). All AF ablation procedures were performed at a single center (Ordensklinikum Linz Elisabethinen) by one out of five experienced interventional cardiologists. There were no specific patient-related exclusion criteria, yet only patients ablated with the CARTO mapping and QDOT MICROTM ablation system were included. The decision between the two possible ablation strategies was at the discretion of the treating interventional electrophysiologist. AF ablation was performed according to the HPSD protocol with the standard ablation catheter (QDOT MICROTM; Biosense Webster, Irvine, California, USA). Values of left ventricular ejection fraction (LVEF) and left size were retrieved from standardized transthoracic echocardiographic examinations usually performed before AF ablation. 

### 2.2. Atrial Fibrillation Ablation Procedure

All patients underwent either contrast-enhanced cardiac computed tomography with cardiac segmentation or transesophageal echocardiography to exclude intracardiac thrombi prior to ablation. An uninterrupted anticoagulation scheme with direct anticoagulants was used, while for patients on vitamin-K antagonists, the target international normalized ratio was between 2–3. During the procedure, unfractionated heparin was continuously administered to achieve an activated clotting time above 300 s. All procedures were performed under continuous sedation using midazolam, disoprivan and fentanyl. After 3-time venous access, a steerable diagnostic catheter was placed into the coronary sinus. In all patients, a single transseptal puncture with double access to the LA using a steerable 11.7-French sheath was performed (Agilis, Abbott, Minneapolis, MN, USA). Mapping was performed using a three-dimensional (3-D) electroanatomic mapping system (CARTO 3, Biosense Webster, Diamond Bar, CA, USA) in combination with a 20-electrode circular mapping catheter (LASSO; Biosense Webster, Diamond Bar, CA, USA) or in selected patients with a multipolar catheter (PENTARAY; Biosense Webster, Diamond Bar, CA, USA). For PVI, a QDOT MICROTM catheter (Biosense Webster, Irvine, CA, USA) in conjunction with a nGEN generator (Biosense Webster, Irvine, CA, USA) was used. According to the CLOSE protocol, an interlesion distance of 6 mm was aimed for both groups [7]. In the very HPSD cohort, RF energy was applied with 90 Watts over the period of 4 s per lesion, while RF energy was applied in an Ablation Index-guided manner in the 50 Watt cohort (AI 400 for posterior wall ablation, AI 550 for anterior wall ablation). Target contact force was set between 10 and 40 g at all ablation sites for both groups. Complete wide antral PVI was performed using a point-by-point ablation technique. Confirmation of effective PVI was performed with a 20-electrode circular mapping catheter placed into each PV ostia by proof of entry/exit block or by proof of exit block while pacing along the ablation line using the ablation catheter. In case of a missed first pass isolation, additional ablation lesions were applied at the earliest PV activation time on the level of the circumferential lesion in order to achieve complete PVI. The presence of pericardial effusion was checked with transthoracic echocardiography at the end of the procedure. 

### 2.3. Follow-Up/Study Outcomes

Procedural efficacy was evaluated using electrocardiogram patch Holter devices (Borsam biomedical instruments CO, Xili Town, Shenzhen, China) over a five-day surveillance period which were sent to the patients by mail at least one year after the AF ablation procedure in combination with the AFEQT questionnaire. AF recurrence was defined as a heart rhythm with no discernible repeating P waves and irregular RR intervals (when atrioventricular conduction is not impaired) of equal or more than 30 s in the patch Holter-ECG tracing. In case of symptoms potentially related to a potential AF recurrence episode, an urgent in-house follow-up, including an ECG recording, was ordered. If the standard 12-lead ECG recording showed AF, this was counted as AF recurrence.

### 2.4. Assessment of Patients’ Health Status

The Atrial Fibrillation Effect on Quality of Life (AFEQT) questionnaire was administered to all patients during the 1-year follow-up by mail. The AFEQT is a 20-item questionnaire that quantifies four domains of AF-related QoL, including symptoms, daily activities, treatment concerns, and treatment satisfaction, using 7–10-point Likert response scales [8]. An overall summary (OS) score can be calculated from the first three domains and ranges from 0–100, where 0 represents the most severe symptoms, physical limitations, and treatment concerns, and 100 represents the best AF-specific health status. A previous study comparing the EHRA symptom classification in AF and AFEQT showed that the mean AFEQT-OS score in patients classified as EHRA class 1 (e.g., no symptom) was 78.4 (SD 19.0) [9]. Thus, we regarded patients with AFEQT-OS scores ≥ 80 as those with preserved HR QoL and patients with AFEQT-OS scores < 80 as those with impaired HR-QoL. A culturally and linguistically translated version of the AFEQT for Austria was used.

### 2.5. Statistical Analysis

Quantitative data are presented as mean ± standard error of the mean (SEM) and were compared using the Student’s t-test for normally distributed data. Qualitative data are presented as absolute and relative frequencies and compared using the Chi^2^ test or the Fisher’s exact test, as appropriate. To evaluate the prognostic value of predefined variables on the study endpoints, Kaplan–Meier curves were calculated with log-rank testing for statistical significance. The result of a statistical test was considered significant for *p* < 0.05; *p* values ≤ 0.1 were defined as a statistical trend. SPSS (Version 24, IBM Armonk, New York, NY, USA) was used for statistics.

## 3. Results

### 3.1. Study Population

**Figure 1:**
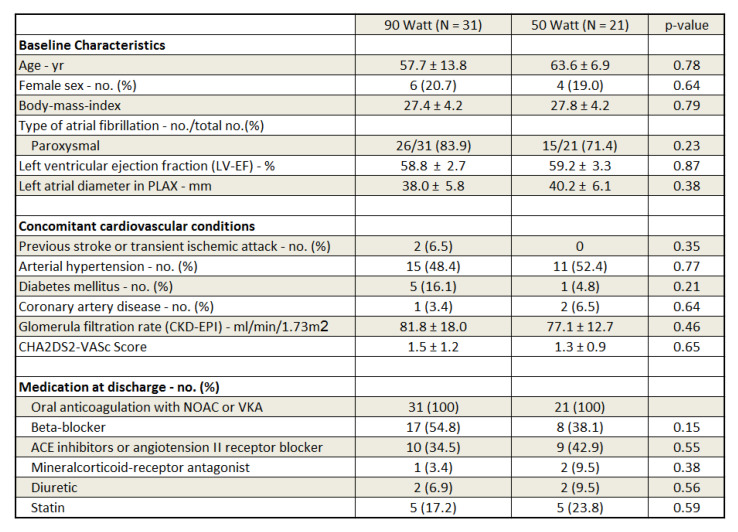
Patient characteristics at baseline.

Between July 2020 and December 2020, including the 9 patients from May 2018, 52 patients (60 ± 11 years; 81% male; 80% paroxysmal AF) underwent a first AF ablation procedure using HPSD protocol at our institution. Out of those, 31 (58 ± 14 years; 79% male; 84% paroxysmal AF, BMI 27.4 ± 4.2 kg/m^2^) were ablated using the 90 Watt 4 s very HPSD protocol. In comparison, 21 patients (64 ± 7 years; 81% male; 71% paroxysmal AF, BMI 27.8 ± 4.2 kg/m^2^) were treated with the 50 Watt HPSD-CLOSE approach. Between both groups no statistically significant differences within the baseline characteristics could be found (age: *p* = 0.78; gender distribution: *p* = 0.64; type of atrial fibrillation: *p* = 0.23, BMI: *p* = 0.79). The mean left atrial diameter derived from the transthoracic echocardiographic parasternal long-axis was 38 ± 6 mm in the very HPSD group and 40 ± 6 mm in the 50 Watt HPSD-CLOSE cohort (*p* 0.38). Among both groups, no statistically significant differences within the concomitant cardiovascular disease were observed (percentage of arterial hypertension was 48% vs. 52%, *p* = 0.77; percentage of diabetes mellitus was 16% vs. 5%, *p* = 0.21; percentage of previous stroke or transient ischemic attack was 6.5% vs. 0%: *p* = 0.35, percentage of coronary artery disease was 3% vs. 7%, *p* = 0.64, glomerular filtration rate (CKD-EPI) 82 ± 18 mL/min/1.73 m^2^ vs. 77 ± 13 mL/min/1.73 m^2^, *p* = 0.46). Consecutively, the CHA2DS2-Vasc-Score was comparable (1.5 ± 1.2 vs. 1.3 ± 0.9; *p*= 0.65). The medication at discharge was without any statistically significant differences (see Figure 1 for detailed patient characteristics).

### 3.2. Atrial Fibrillation Ablation Procedure

**Figure 2:**
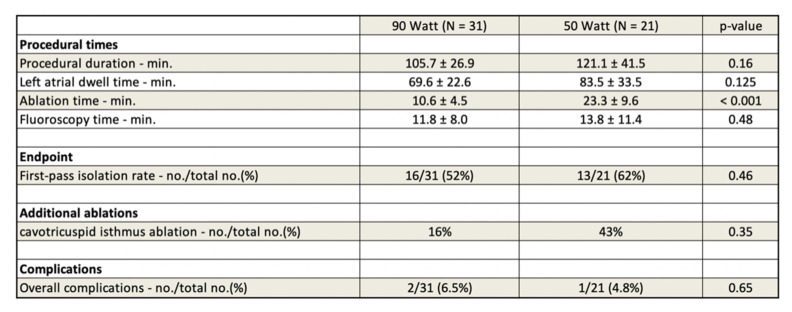
Procedural data.

**Figure 3:**
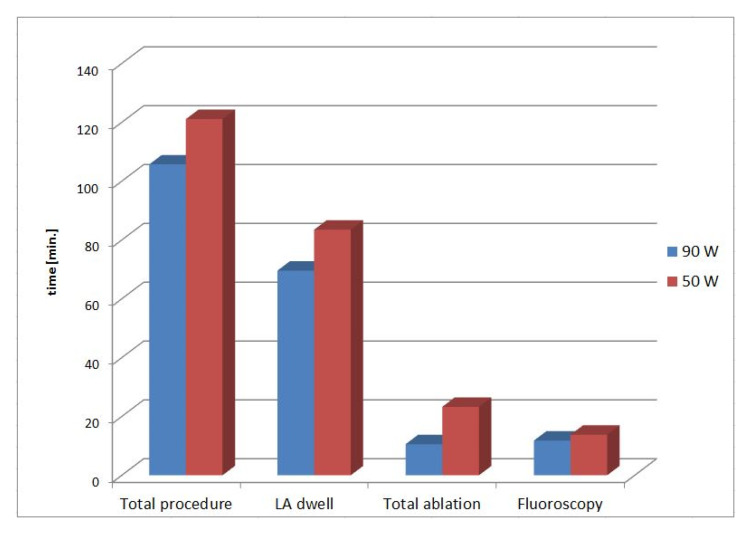
Comparison of procedural times between both ablation protocols.

Overall measured procedural (105.7 ± 26.9 min vs. 121.1 ± 41.5 min; *p* = 0.16), left atrial dwell times (69.6 ± 22.6 min vs. 83.5 ± 33.5 min; *p* = 0.125), and fluoroscopy times (11.8 ± 8.0 vs. 13.8 ± 11.4 min; *p* 0.48) showed non-significant trends of time-saving for the very HPSD group with the expected highly significant reduction in ablation time (10.6 ± 4.5 min vs. 23.3 ± 9.6 min; *p* < 0.001). There was no significant difference concerning the first-pass isolation rate (62% vs. 52%, *p* =0.46) as the complication rate (very HPSD group n = 2: 1 × pseudoaneurysm with the need of thrombin-injection and 1 × pericardial effusion with the need of pericardial puncture; HPSD-CLOSE group n = 1: pericardial effusion with the need of pericardial patch repair; *p* = 0.65) compared to the HPSD CLOSE approach group. Additional ablations of the right atrial cavotricuspid isthmus for documented typical atrial flutter were performed in 14 patients (16% vs. 43%, *p*= 0.35). (See Figure 2 and Figure 3 for detailed procedural characteristics).

### 3.3. Short and Long-Term Outcomes

**Figure 4:**
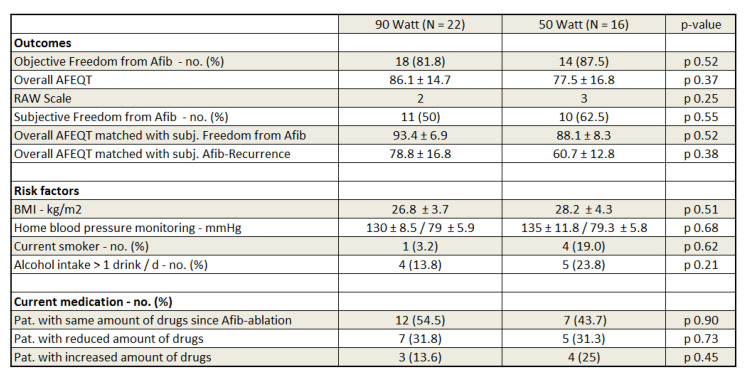
Long-term follow-up data.

**Figure 5:**
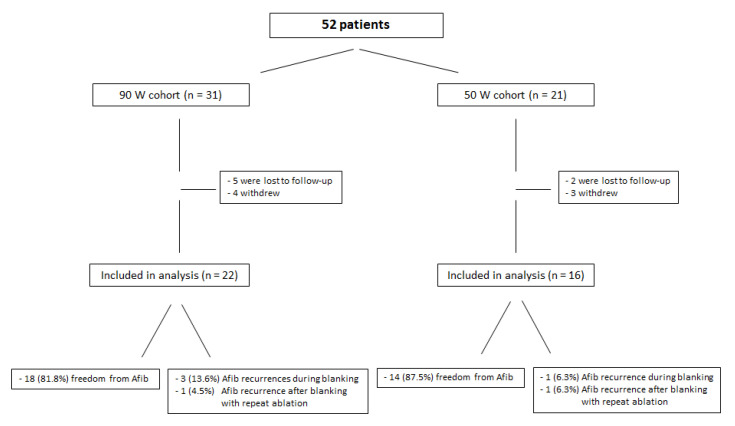
Detailed flow-chart overview of study population.

**Figure 6:**
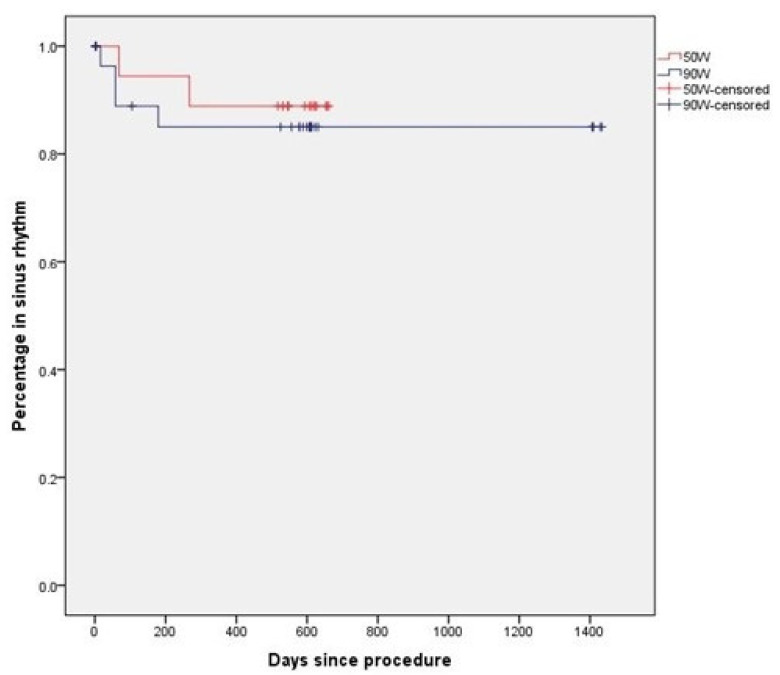
Kaplan Meier Curve outcome analysis- days from intervention to first Afib recurrence.

Loss of follow-up was 16.1% vs. 9.5%, *p*. 0.40. Four out of thirty-one (12.9%) patients in the very HPSD group withdrew consent vs. three out of twenty-one (14.3%) in the 50 Watt HPSD group, *p*. 0.60. The median follow-up interval was 23.2 ± 9.9 months (minimum 18 months, maximum 46 months) without a statistical difference between both groups (*p* = 0.46). During the 3 months blanking period, 8% of all patients suffered from AF recurrence (14% vs. 6%; *p* = 0.52). On top of those events, one patient from each group suffered from an AF recurrence (5% vs. 6%, *p* = 0.68) and was scheduled for a redo-procedure in the meantime. At the end of the follow-up interval, freedom from atrial fibrillation was similar in both ablation arms (82% vs. 88%, *p* = 0.52) (Figure 4, Figure 5 and Figure 6).

### 3.4. Health Status Outcomes

At the end of the follow-up interval, patients in the very HPSD cohort had a slightly higher, though statistically non-significant mean AFEQT-OS score compared to the 50 Watt HPSD CLOSE arm (86.1 ± 14.7 vs. 77.5 ± 16.8, *p* = 0.37) with a tendency towards fewer patients with an impaired HR-QoL (AFEQT-OS score < 80) (30.4% vs. 46%, *p* = 0.31). Unexpected and in contradiction to the findings in the Holter-ECG recordings, only around half of our patients, regardless of the ablation protocol, stated subjective freedom from atrial fibrillation (50% vs. 62.5%, *p* = 0.55). After adjusting the AFEQT-OS score within the subgroup of patients who reported the subjective impression of freedom from AF, no difference between both groups could be found (93.4 ± 6.9 vs. 88.1 ± 8.3, *p* = 0.52). The same was true when comparing only the subgroups with the subjective impression of an AF recurrence (78.8 ± 16.8 vs. 60.7 ± 12.8, *p* = 0.38). Only the patients who mentioned a subjective freedom state from atrial fibrillation independent from their ablation protocol to the group of patients with the subjective impression of an AF recurrence led to a highly significant difference in the AFEQT-OS score (90.3 ± 8.0 vs. 75.9 ± 19.3, *p* = 0.006). Risk factors were well controlled within both groups and did not differ significantly between both groups at this time (home blood pressure monitoring: 130 ± 8.5/79 ± 5.9 vs. 135 ± 11.8/79 ± 5.8, *p* = 0.68; BMI: 26.8 ± 3.7 vs. 28.2 ± 4.3 kg/m^2^, *p* = 0.51; percentage of smokers: 3% vs. 19%, *p* = 0.62; >1 standard alcoholic drink per day: 14% vs. 24%, *p* = 0.21). Identically to the baseline, no statistically significant difference in the current medication was seen between both groups (taking the same amount of drugs since the ablation: 54.5 % vs. 43.7%, *p* = 0.90; reducing in the number of drugs: 31.8% vs. 31.3%, *p* = 0.73; taking an increased amount of drugs: 13.6% vs. 25%, *p* = 0.45).

## 4. Discussion

The purpose of our study was to investigate short- and long-term outcome data of patients undergoing first-do-symptomatic PVI with a very HPSD 90 W 4 s ablation protocol compared to a 50 Watt AI strategy. Our results provide hints that very HPSD ablation does significantly shorten ablation times in PVI using RFA. Within our relatively small sample population, we also realized a statistically non-significant tendency towards time-saving within the other measured procedural times (left atrial dwell time, total procedure time and fluoroscopy time). These later findings are similar but not totally in line with the results of Bortone et al., showing that within their cohort, 90 W 4 s PVI ablation led to similar procedural times due to a lower first pass PVI-rate and higher acute PV reconnection rate [10]. The minor differences between the results of those two studies might be caused by the relatively small study population size but might partly be explained by the fact that the 90 W 4 s protocol is much more prone to suboptimal lesion formation in cases of an unstable ablation catheter position during RF-energy application. In our study, the decision as to which protocol should be selected was made after finishing the diagnostic map, already having the ablation catheter placed into the left atrium and having had the chance to obtain an impression on the catheter movement during the respiratory cycle. Taking the previously said together, we do believe that 90 W 4 s PVI ablation does lead to shorter procedural times if applied in selected patients and in experienced hands. Both studies were able to show that the very HPSD ablation protocol is safe without hints of a higher complication rate.

Short-term and long-term success rates were comparable between the two groups, with a first-pass isolation rate of 52% and 62% (*p* =0.46) and a one-year sinus rhythm maintenance of 82% and 88% (*p* = 0.52). Those numbers are in line with Bortone’s cohort [9]. In relation to former studies, the one-year sinus rhythm maintenance percentage seems quite high, especially when comparing them to the significantly lower number of patients who had the subjective impression of complete freedom from AF (50% vs. 62.5%, *p* = 0.55). Taking this into consideration, according to the guidelines, the indication for PVI ablation is still based on symptoms and the subjective presence/recurrence of AF goes hand in hand with a reduced quality of life expressed in the statistically lower AFEQT-OS score (90.3 ± 8.0 vs. 75.9 ± 19.3, *p* < 0.01). Trying to correlate the symptomatic patients to any potentially existing differences in the baseline characteristics as in the risk factor control did not lead to any significant findings. One of the reasons might be that our study cohort was younger and had a CHA2DS2-VASc Score of around 1.5, meaning the patients were less sick than the ones reported in previous studies [3]. Second and probably the most probable reason is that our success numbers are based on a single Holter-ECG recording over the period of five days one year after the index ablation. Proven by numerous studies, a single Holter-ECG recording for the assessment of the efficacy of AF ablation is clearly not ideal [11,12]. However, other surveillance regimes, e.g., daily 30 s ECG transmissions, are often difficult to implement, not only into the patient routine, requiring functioning technical skills, but also in daily clinician business. On the opposing side, the technological advances during the last couple of years, as well as above-average well-controlled risk factors, might have influenced our results beneficially.

## 5. Limitations

Our study has several potential limitations. First, its non-randomized observational nature is associated with inherent limitations. The results of our study may not be transferrable to other centers using different ablation protocols, catheters or settings.

Due to the small sample size in each group, drawing definite conclusions about the two different ablation techniques would be premature. In comparison to other trials, the prevalence of age-associated comorbidities was lower. We did not calculate the AFEQT-OS score at baseline, making it harder to draw conclusions from the values during follow-up. As mentioned before, a single Holter-ECG recording most likely leads to overestimating the success from the ablation by missing some of the AF recurrences. Adding the nine patients from the QDOT-FAST trial to the 90 Watt very HPSD group is a clear limitation of the study because of the long interval between the ablation date and the follow-up, considering that our follow-up data are already quite inhomogeneous. Although very HPSD ablation is expected to reduce the risk of collateral tissue damage such as esophageal thermal injury (EDEL), asymptomatic EDEL may have occurred in the studied population due to the lack of gastrointestinal endoscopic evaluation. Furthermore, the occurrence of asymptomatic cerebral infarction was not assessed in this study.

## 6. Conclusions

In contemporary practice, very HPSD (90 W 4 s) ablation seems to be safe and effective for achieving freedom from atrial fibrillation recurrences. We found hints that it also shortens ablation times with a tendency towards time-saving within the other measured procedural times if applied in selected patients and experienced hands.

## Data Availability

The data underlying this article will be shared on reasonable request to the corresponding author.
